# Recovery from Apparent Death after Exposure to the Vapour of Charcoal

**Published:** 1830-06

**Authors:** M. Rene Bourgeois


*Recovery from apparent Death after Exposure to the Vapour of Charcoal.* By M. RENE BOURGEOIS, D.M.P. &c. (Condensed from the *Archives Gen.*)

A SERVANT boy was found by his master in bed, entirely motionless and senseless. It was supposed that the lad had died suddenly, but M. B. was immediately sent for. He detected, on entering the room, a slight odour of charcoal, and thought it probable that the patient was in a state of asphyxia, from exposure to mephitic air. He was confirmed in this opinion by perceiving a night lamp on a table no longer burning, although it was well supplied with oil, and an iron pan in which were vestiges of half-burnt charcoal. Without waiting to make further inquiries, and notwithstanding the weather was intensely cold, M. B. did not hesitate to remove the boy to the middle of an open yard, where he was placed, almost uncovered and as upright as possible, in a chair.

A careful examination was now made, and the patient was found to be in the following state: Limbs flaccid and motionless, and, like the head, following automatically all the movements of the body; heat of a natural degree, and equably distributed over the surface; mouth open; pupils dilated and fixed; the lips and upper eyelids slightly swollen, and of a bluish colour; neither any respiratory movement nor pulsation of the heart or arteries was to be detected; the urine passed away by drops, the feces had been discharged; general insensibility. In a word, the boy was to all appearance dead.

Although, under such circumstances, but little was to be hoped from medical assistance, M. B. determined to make every effort to restore animation. After exposure to the air for more than half an hour, during which time cold vinegar and water was frequently dashed upon the head and face, whilst ether was occasionally applied to the nostrils, the patient was removed into a large room, and placed upon a camp bedstead, in such a situation that he was exposed to a free current of air. The windows were all opened, and only two assistants retained in the apartment, who, with M. B., briskly rubbed dry the surface of the whole body with flannel, after sprinkling it with ether and vinegar. In a careful manner, and at different intervals, air was blown into the respiratory passages, either with the mouth or a pair of bellows. From the fear that the mephitic gas which filled the bronchia might, from its density, impede the entrance of a current of air impelled in either of the above modes, M. B. introduced into the mouth, near the opening of the glottis, the extremity of an exhausting syringe, by the action of which he endeavoured to effect the purposes of expiration. A large number of brimstone matches were lighted, and then, having formed a fumigation with salt and sulphuric acid, the vapour was directed towards the mouth and nostrils. Several clysters were administered of cold vinegar and water. Although there was a purple turgescence of the countenance, which indicated a congestive state of the subcutaneous vessels, venesection was not had recourse to, as there was as yet no proof of the continuance of the circulation of the blood. To ascertain this point, a ligature was placed upon the arm and gradually tightened, but none of the superficial vessels became enlarged in consequence. Cupping glasses were applied, but no blood could be drawn.

All these various means had been pursued for a long time without any appreciable result, when M. B., on applying his ear to the chest of the patient, fancied he heard at intervals, near the junction of the bronchia with the trachea, a sort of guggling noise, similar to that which arises from a small volume of air rapidly passing through a collection of mucus, but each time the sound was so weak and momentary that it could not be very clearly heard. Still the hopes of the attendants were excited. After some time the motion of flatus was distinctly heard in the intestines; and, although this motion was possibly passive and insignificant, M. B. was willing to persuade himself that it was a proof of returning irritability in the intestinal canal. The stethoscope was now constantly applied to the chest, and interrogated with great anxiety. At last, about eight o'clock in the morning, three hours from the arrival of M. B., a very slight *râle* in the trachea indicated the first expiration. The sound was so indistinct that its reality was doubtful, until a clear mirror that was applied to the mouth was found sullied with the breath. Almost at the same moment a feeble contractile motion of the nostrils was perceived, accompanied by a slight sound from the exit of air. A frothy matter was now occasionally thrown from the mouth, and an irregular hiccup came on, which was followed by a sort of horripilation and trembling of the surface of the body. A ligature was again applied to the arm, and the veins below it now became swollen and tense. Eight ounces of very thick black blood were drawn with some difficulty. Ligatures were successively applied to the ankles, the hams, the calves of the legs, and upon the thighs and arms, as M. B. imagined that a stimulus might be afforded to a more vigorous circulation of the blood, by causing distention of the vessels by a ligature, and then suddenly removing it. The frictions were assiduously continued, and strong sinapisms were applied to the inferior extremities. Cupping glasses were also applied to the chest and along the spine. The respiratory movements gradually became more distinct; the mucous *râle*, which could at first only be detected by the stethoscope, was now heard at some distance. In a short time the breathing was loud and stertorous; the circulation was gradually reestablished, and the pulse became nearly of a natural character.

Although the organic functions appeared to be restored, the patient remained in a state of profound coma, motionless and perfectly insensible. M. B., however, felt confident that he should succeed in restoring the patient, and, after *eleven hours'* anxious attendance, he ventured to leave him for three quarters of an hour. Upon his return he found the boy, much to his surprise, with his eyes open, and in perfect possession of his senses, talking loudly with a number of persons who had been drawn to the spot by the report of his "resurrection."

When he revived, his first belief was that he had overslept himself, and he was anxious to open the shop without loss of time. It was with difficulty that he was induced to believe what had occurred: but he remembered that he went to bed at ten o'clock the night before, and, being cold and with his feet wet, he filled an iron pan with lighted charcoal, in order to dry himself, and that he had left it burning.

To the detail of this interesting case, M. B. adds some good practical remarks. With respect to the treatment of similar instances of asphyxia, he observes, that the first thing to be done is to expose the patient to a cool and dry air. Air is then to be introduced into the lungs by means of a laryngeal tube: M. B. would not rely upon the mouth of an assistant or a pair of bellows to effect this very important object. It would appear, from the recent experiments of M. Leroy d'Etiolles and other physiologists, that the practice of insufflation is not unattended with danger; and M. B. admits the impropriety of it when the lungs possess sufficient irritability to react against the violent admission of air; but he doubts whether the arguments of Leroy d'Etiolles will apply, if insufflation be only practised when the function of respiration is completely abolished, which is indeed the only case which requires such an expedient. He also suggests the probable advantage of first emptying the respiratory passages of the vitiated air which they contain, which he attempted to do in the above case by means of the exhausting syringe. Dry frictions briskly applied over the whole surface of the body should be promptly employed. M.B. coincides with Segalas in the opinion that, besides the direct stimulus thus afforded to the nervous fibrils of the skin, the friction produces, by promoting absorption, a kind of cutaneous respiration; a commencement of oxygenation of the blood contained in the superficial capillaries. Cupping glasses should also be applied, with and without scarification. M. B. does not conceive that general bleeding is so strongly indicated in such instances as the above, as in those cases of asphyxia which depend upon sudden violence, and in which some organ essential to life is suddenly overloaded by an overwhelming congestion of blood. In the former case there is no true congestion, but rather a general and passive stasis of a column of black blood, for the circulation of which the accustomed actions of the heart are alone required. It is now satisfactorily ascertained that an imperfect and slow circulation of the blood may go on in the large vessels after the heart has ceased to pulsate. The swelling of the veins in the subject of the above case from the application of ligatures around the limbs, is a proof of this fact.

In conclusion, M. René Bourgeois directs attention to the efficacy of appropriate treatment in such cases, when it is employed with due perseverance, even when there is apparently little or no hopes of success. In the above instance, three hours elapsed before any signs of vitality were detected, and the patient did not regain his consciousness for twelve hours. We should not, therefore, abandon such cases until the most unequivocal signs of death are present, and still less ought the burial of the body to be hastened. We should bear in mind the adage so well expressed by the inimitable Molière:

> "Qui tôt ensevelit bien souvent assassine,
> Et tel est cru défunt qui n'en a que la mine."

The persevering kindness with which M. René Bourgeois continued his efforts to restore animation in the above case is alike creditable to his professional and philanthropic feeling. His success, under circumstances which might have made many practitioners despair, affords additional evidence of the necessity for the most determined continuance of appropriate treatment in cases of suspended animation. However frequently practitioners have been warned against the hasty abandonment of patients in a state of asphyxia, we still fear that lives are not unfrequently sacrificed, which might be saved by the exertion of the same degree of zeal and perseverance which reflect so much honour upon the relator of the above case.

